# Data on growth and production of (*Aloe vera* L.) treated by different levels of vermicompost and nitrogen fertilizer

**DOI:** 10.1016/j.dib.2018.12.052

**Published:** 2018-12-20

**Authors:** Fatemeh Nejatzadeh

**Affiliations:** Department of Horticulture, Faculty of Agriculture, Islamic Azad University of Khoy, Khoy, Iran

**Keywords:** Vermicompost, Nitrogen, Sucker, *Aloe vera*

## Abstract

Data on the effect of vermicompost and nitrogen on growth and production of *Aloe vera*, an experiment was carried out in 2015–2016 at Faculty of Agriculture of Islamic Azad University of Khoy. This experiment was arranged as factorial, based on RCB design with four replications. Treatments were 4 levels of vermicompost (Control, 75, 150 and 200 gr per pot) and four levels of nitrogen fertilizer (N1: control, N2: 500, N3: 1000, and N4: 1500 mg per pot). Nitrogen fertilizer was split into three stages (8 leaves, before suckering and the beginning of formation suckers). Traits investigated such as plant height, number of leaves, stem diameter, number of suckers, number of leaf sucker, sucker weight, sucker height and total biomass. This article present data regarding the application of 150 g of vermicompost and 1000 mg of nitrogen and 200 g of vermicompost and 1500 mg of nitrogen on the characteristics of sucker. Therefore, data on simultaneous application of vermicompost and nitrogen fertilizer in *Aloe vera* cultivation as an important role in the production and enhancement of sucker and plant yield mentioned.

**Specifications table**

**Table:** 

Subject area	*Chemistry, biology*
More specific subject area	Effect of Vermicompost and Nitrogen on Growth and Production of *Aloe vera*
Type of data	*Table and figure*
How data was acquired	*Laboratory equipments such as Ruler, caliper, Digital Balance*
Data format	*Analyzed data*
Experimental factors	Effect of Vermicompost and Nitrogen on Growth and Production of *Aloe vera*
Experimental features	*Treatments were 4 levels of vermicompost (Control, 75, 150 and 200 gr per pot) and four levels of nitrogen fertilizer (N1: control, N2: 500, N3: 1000, and N4: 1500 mg per pot). Nitrogen fertilizer was split into three stages (8 leaves, before suckering and the beginning of formation suckers). In June, uniform suckers with a size of 18 to 20 cm were randomly selected and transferred to the greenhouse, and in pots with capacity of 20 kg of soil was planted. Before planting vermicompost treatments were added to pots and completely mixed with the soil. The greenhouse temperature for the growth of the Aloe vera was 28 °C/ day and 22 °C at night uniformly. Plants were irrigated on the crop capacity. At the end of the plant growth period, traits such as plant height, number of leaves, stem diameter, number of suckers, number of leaf sucker, sucker weight, sucker height and total biomass were measured.*
Data source location	*Islamic Azad University of Khoy, Iran*
Data accessibility	*All data are present in this article*
Related research article	– Analysis of Different Levels of Superabsorbent and Vermicompost Effects on Performance and Aloe Vera Operation Component In Yasouj Region, Research Journal Of Fisheries And Hydrobiology, 10(9) May 2015, Pages: 573–580 – Effect Of Vermicompost and Compost on Lettuce Production, Chilean Journal of Agricultural Research. 2010 - Vol. 70 - N 4. – Lettuce (Lactuca sativa ‘Webb׳s Wonderful’) shoot and root growth in different grades of compost and vermicomposted compost. Acta Horticulture. 2016, 1146, 33–40. – Comparative efficacy of vermicomposted paper waste and inorganic fertilizer on seed germination, plant growth and fruition of Cyamopsis tetragonoloba. Journal of Applied Horticulture. 2014, 16, 40–45. – Effect of macrophyte vermicompost on growth and productivity of brinjal (Solanum melongena) under field conditions. Int. J. Recycl. Org. Waste Agric. 2015, 4, 73–83. – Effect of Vermicompost and Chemical Fertilizer on Growth and Yield of Hyacinth Bean, Lablab purpureus (L.) Sweet. Dynamic Soil, Dynamic Plant, 2008, 2 (2), 77–81

**Value of the data**

• The data show the significant effect of Vermicompost on plant height, number of leaves, stem diameter, number of sucker, stem weight, number of leaf sucker, height of sucker and total biomass of *Aloe vera*
• The data indicate Vermicompost and Nitrogen treatments caused beneficial in improving Growth and yield of *Aloe vera* suckers
• The data highlight positive effect of vermicompost and nitrogen on growth and production of suckers in *Aloe vera*.
• These data may be relevant for (i) other researchers using Vermicompost and Nitrogen in their experiments and (ii) for further research that focuses on the increasing of yield of *Aloe vera*

## 1 Data

**Fig. 1:**
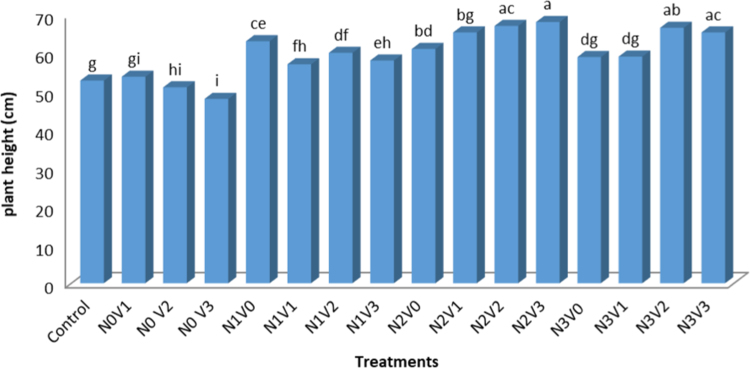
Interaction between vermicompost and nitrogen fertilizer on plant height in *Aloe vera,* In each column, the same letters indicate that there is no significant difference between the meanings (LSD test).

**Fig. 2:**
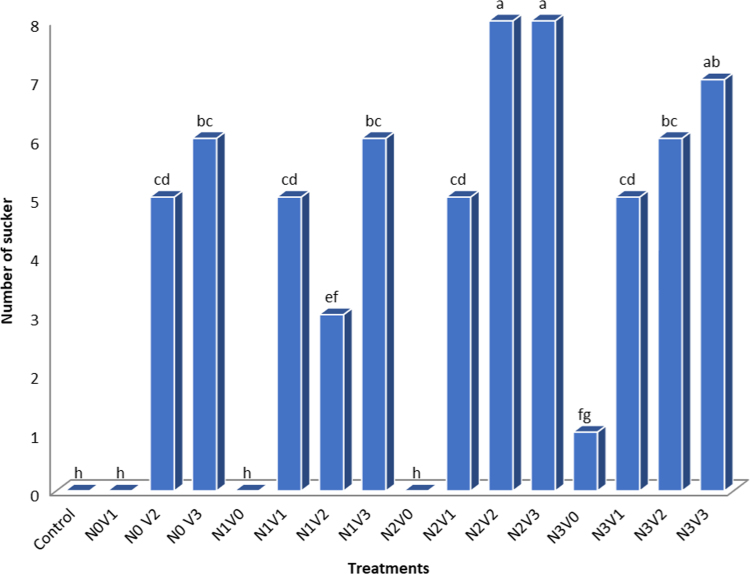
The Effect of Vermicompost and Nitrogen Fertilizer on Number of sucker in *Aloe vera,* In each column, the same letters indicate that there is no significant difference between the meanings (LSD test).

**Fig. 3:**
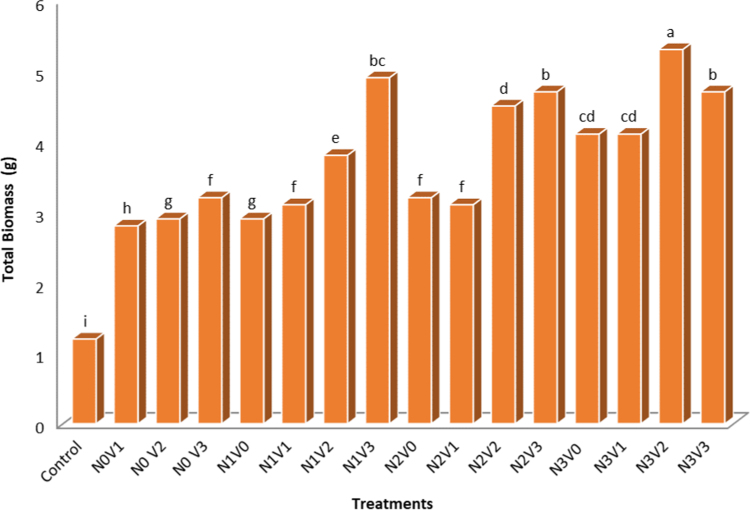
The Effect of Vermicompost Fertilizer and Nitrogen on Total Biomass in *Aloe vera,* In each column, the same letters indicate that there is no significant difference between the meanings (LSD test).

Below, reported the effects of Vermicompost and Nitrogen on Growth and Production of *Aloe vera*. The result of Physic-Chemical analysis of the soil are present at Table 1. Physical and chemical properties of vermicompost used in the experiment are shown in Table 2. Analysis of variance of vermicompost and nitrogen on studied traits in *Aloe vera* are present in Table 3. Comparison of mean vermicompost and nitrogen treatments on the traits of *Aloe vera* are shown in Table 4. Comparison of the mean of interaction between vermicompost and nitrogen on studied traits in *Aloe vera* are present in Table 5. Simple correlation coefficients of the traits studied in the *Aloe vera* are shown in Table 6. Interaction between vermicompost and nitrogen fertilizer on plant height in *Aloe vera* are depicted in Fig. 1 The Effect of Vermicompost and Nitrogen Fertilizer on Number of sucker in *Aloe vera* are shown in Fig. 2*. The Effect of Vermicompost Fertilizer and Nitrogen on Total Biomass in Aloe vera* are depicted in Fig. 3.

**Table 1:** The result of Physic-Chemical analysis of the soil.

Texture	Lime	Percent Saturated	Organic carbon (%)	Total N (%)	Available K ppm))	Available P	EC (ds m-1)	pH
Clay	30.74	60.98	2.57	0.25	314	8.43	1.57	8.66

**Table 2:** Physical and chemical properties of vermicompost used in the experiment.

WSC	CEC	W	Na	Fe	Mn	Zn	Cu	Mg	Ca	K	P	N	Ash	N.A	OC%	EC(ds m-1)	pH
–	–	–	0.02	0.38	0.89	2.5	0.26	0.38	1.95	0.79	0.21	0.98	25	0.01	8	1.97	6.6

**Table 3:** Analysis of variance of vermicompost and nitrogen on studied traits in *Aloe vera.*

Source of variations plant height (cm)	df	Means of squares							
		Total biomass (kg)	sucker height (cm)	number of leaf sucker	sucker weight (g)	number of Sucker	stem diameter (cm)	number of leaves	
Replication	3	ns 0.030	ns 4.40	ns 0.40	ns 41.476	1.27	ns 14.61	1.2	ns 46.12
Vermicompost	3	8.45**	1048.85**	57.38**	2489.43**	108.81**	73.34**	32.70**	155.45**
Nitrogen	3	20.49**	538.45**	45.76**	2063.41**	5.74**	28.61**	7.17**	618.7**
V× N	9	0.48**	136.08**	13.86**	525.90**	11.97**	ns 18.7	1.16*	78.47**
Error	32	0.0478	4.8	0.28	43.76	0.82	9.26	0.46	18.34
CV (%)	–	5.76	15.74	12.40	25.28	23.02	6.68	3.1	6.93

ns, * and **: Non-significant and significant at the 5 and 1% levels of probability, respectively.

**Table 4:** Comparison of mean vermicompost and nitrogen treatments on the traits of *Aloe vera.*

Treatment	Traits studied								
	Level of treatment	Total biomass (kg)	sucker height (cm)	number of leaf sucker	sucker weight (g)	number of Sucker	stem diameter (cm)	number of leaves	plant height (cm)
Nitrogen	0	2.29 d	7.07 c	2.39 c	10.48 c	3.30 b	41.93 b	20.58 c	52.76 c
	500	3.70 c	14.60 b	4.26 b	27.50 b	3.56 b	45.24 ab	21.30 b	59.57 b
	1000	4.11 b	13.32 b	4.28 b	29.01 b	4.45 a	46.92 a	21.81 a	66.50 a
	1500	5.09 a	21.20 a	6.53 a	36.62 a	4.45 a	46.46 a	22.13 a	65.60 a
Vermicompost	0	3.06 d	3.95 d	1.68 c	9.4 d	0.29 d	43.82 b	20.17 b	61.75 a
	75	3.34 c	11.92 c	4.39 b	24.79 c	4.08 c	43.73 b	20.28 b	57.30 b
	150	4.09 b	17.30 b	5.58 a	32 b	5.11 b	48.16 a	22.80 a	64.51 a
	200	4.62 a	22 a	6.80 a	38.42 a	6.32 a	46.34 a	20.59 a	63.06 a

In each column, the same letters indicate that there is no significant difference between the meanings (LSD test).

**Table 5:** Comparison of the mean of interaction between vermicompost and nitrogen on studied traits in *Aloe vera*.

Treatment	Sucker height (cm)	Number of leaf sucker	Sucker weight (g)	Leaf diameter (cm)	Number of leaves
N0V0(control)	0 h	0 g	0 g	40.16 e	17.84 h
N0V1	0 h	0 g	0 g	42.40 cd	20 g
N0 V2	12.72 fg	4.22 f	15.1 f	49.11 a	22.23 bc
N0 V3	15.51 ef	5.29 ce	26.71 e	43.18 bd	22dc
N1V0	0 h	0 g	0 g	42.90 cd	20.23 fg
N1V1	9.94 g	4.4 ef	42.5 ab	44.13 bd	20.23 fg
N1V2	14.4 f	6.05 ac	33.6 ce	48.16 ab	22.23 bc
N1V3	33.22 a	6.26 ab	35.03 be	45.68 ac	22.41 bc
N2V0	0 h	0 g	0 g	47.93 ab	21.23 de
N2V1	15.43 f	6 ac	29.96 de	45.26 ac	20.64 fg
N2V2	19.1 cd	5.73 bd	43.50 ab	45.70 ac	23.60 a
N2V3	18.4 de	5.25 de	42.55 ab	47.51 ab	23 ab
N3V0	15.85 ef	6.70 a	37.4 bd	44.20 bd	21 ef
N3V1	22.30 bc	6.76 a	27.02 e	43.05 cd	20.23 fg
N3V2	22.68 b	6.26 ab	36.2 bd	49.4 a	23 ab
N3V3	23.93 b	6.30 ab	49.26 a	48.93 a	23 ab

In each column, the same letters indicate that there is no significant difference between the meanings (LSD test).

**Table 6:** Simple correlation coefficients of the traits studied in the *Aloe vera*.

	Plant height	Number of leaves	Stem diameter	Number of sucker	Sucker weight	Number of leaf sucker	Sucker height	Total biomass
Plant height	1							
Number of leaves	0.677**	1						
Stem diameter	0.677**	0.722**	1					
Number of sucker	ns 0.182	0.621**	ns 0.480	1				
Sucker weight	ns 0.377	0.652**	ns 0.431	0.711**	1			
Number of leaf sucker	ns 0.257	0.587*	ns 0.408	0.777**	0.884**	1		
Sucker height	ns 0.312	0.658*	ns 0.424	0.757*	0.806**	0.888**	1	
Total biomass	0.624**	0.718**	0.571*	0.572**	0.810**	0.800**	0.841**	1

ns, * and **: Non-significant and significant at the 5 and 1% levels of probability, respectively.

## 2 Experimental design, materials, and methods

An experiment was conducted to evaluate the effect of vermicompost and nitrogen on growth and production of *Aloe vera*. Treatments were 4 levels of vermicompost (Control, 75, 150 and 200 gr per pot) and four levels of nitrogen fertilizer (N1: control, N2: 500, N3: 1000, and N4: 1500 mg per pot) [1], [2]. Nitrogen fertilizer was split into three stages (8 leaves, before suckering and the beginning of formation suckers). In June, uniform suckers with a size of 18 to 20 cm were randomly selected and transferred to the greenhouse, and in pots with capacity of 20 kg of soil was planted. Before planting vermicompost treatments were added to pots and completely mixed with the soil [3]. The greenhouse temperature for the growth of the *Aloe vera* was 28 °C/day and 22 °C at night uniformly. Plants were irrigated on the crop capacity. The greenhouse experiment was conducted at the Faculty of Agriculture of Islamic Azad University of Khoy in 2015–2016. This experiment was arranged as factorial, based on RCB (Randomized Complete Block) design with four replications. At the end of the plant growth period, traits such as plant height, number of leaves, stem diameter, number of suckers, number of leaf sucker, sucker weight, sucker height and total biomass were measured [4], [5]. Traits such as number of leaves and height of plant were measured by a ruler from the crown (from leaf to leaf tip), for measuring the stem diameter, a digital caliper was used and reported as a centimeter, to measure the traits suckers, suckers were separated from the mother plant and their number was counted and then the height of the suckers, weights of sucker, number of suckers leaves and total biomass of the plant were measured.

### 2.1 Statistical analysis

Analysis of variance of the data was carried out using SAS software. LSD test was applied to compare means of each trait at *p* ≤ 0.05. EXCEL software was used to draw figures.
